# Bedaquiline and linezolid resistance in people with rifampicin-resistant tuberculosis in the Western Cape Province of South Africa

**DOI:** 10.21203/rs.3.rs-9144949/v1

**Published:** 2026-03-18

**Authors:** Erick Auma, Yonas Ghebrekristos, Greshan Kisten, Tiana Schwab, Sarishna Singh, Christoffel Opperman, Janre Steyn, Brigitta Derendinger, Nabila Ismail, Rouxjeane Venter, Robin Warren, Grant Theron

**Affiliations:** DSI-NRF Centre of Excellence for Biomedical Tuberculosis Research, and SAMRC Centre for Tuberculosis Research, Division of Molecular Biology and Human Genetics, Faculty of Medicine and Health Sciences, Stellenbosch University, Tygerberg, Cape Town, South Africa; National Health Laboratory Service, Greenpoint Tuberculosis Laboratory, Cape Town, South Africa.; National Health Laboratory Service, Greenpoint Tuberculosis Laboratory, Cape Town, South Africa.; DSI-NRF Centre of Excellence for Biomedical Tuberculosis Research, and SAMRC Centre for Tuberculosis Research, Division of Molecular Biology and Human Genetics, Faculty of Medicine and Health Sciences, Stellenbosch University, Tygerberg, Cape Town, South Africa; National Health Laboratory Service, Greenpoint Tuberculosis Laboratory, Cape Town, South Africa.; National Health Laboratory Service, Greenpoint Tuberculosis Laboratory, Cape Town, South Africa.; DSI-NRF Centre of Excellence for Biomedical Tuberculosis Research, and SAMRC Centre for Tuberculosis Research, Division of Molecular Biology and Human Genetics, Faculty of Medicine and Health Sciences, Stellenbosch University, Tygerberg, Cape Town, South Africa; DSI-NRF Centre of Excellence for Biomedical Tuberculosis Research, and SAMRC Centre for Tuberculosis Research, Division of Molecular Biology and Human Genetics, Faculty of Medicine and Health Sciences, Stellenbosch University, Tygerberg, Cape Town, South Africa; DSI-NRF Centre of Excellence for Biomedical Tuberculosis Research, and SAMRC Centre for Tuberculosis Research, Division of Molecular Biology and Human Genetics, Faculty of Medicine and Health Sciences, Stellenbosch University, Tygerberg, Cape Town, South Africa; DSI-NRF Centre of Excellence for Biomedical Tuberculosis Research, and SAMRC Centre for Tuberculosis Research, Division of Molecular Biology and Human Genetics, Faculty of Medicine and Health Sciences, Stellenbosch University, Tygerberg, Cape Town, South Africa; DSI-NRF Centre of Excellence for Biomedical Tuberculosis Research, and SAMRC Centre for Tuberculosis Research, Division of Molecular Biology and Human Genetics, Faculty of Medicine and Health Sciences, Stellenbosch University, Tygerberg, Cape Town, South Africa; DSI-NRF Centre of Excellence for Biomedical Tuberculosis Research, and SAMRC Centre for Tuberculosis Research, Division of Molecular Biology and Human Genetics, Faculty of Medicine and Health Sciences, Stellenbosch University, Tygerberg, Cape Town, South Africa

**Keywords:** bedaquiline, linezolid, tuberculosis, drug resistance

## Abstract

**Introduction:**

South Africa was an early implementer of bedaquiline and linezolid for drug-resistant tuberculosis (TB), however, programmatic capacity for drug susceptibility testing (DST) was not initially available.

**Methods:**

We analysed people with rifampicin-resistant (RR)-TB (n=3138) programmatically tested with Xpert MTB/XDR and bedaquiline and linezolid phenotypic (p)DST in the same episode for diagnosis or treatment monitoring. Data from respiratory specimens collected 01/01/2023–31/12/2024 across six districts in Western Cape, South Africa, were included.

**Findings:**

89% (2799/3138) of people were successfully tested with Xpert MTB/XDR, with 12% (332/2799) fluoroquinolone-resistant. 77% (2423/3138) successfully underwent bedaquiline pDST, with 12% (278/2423) resistant. 84% (232/278) of bedaquiline resistance was in the diagnostic (first) isolate. Of these, 51% (118/232) had no prior DR-TB (45 prior drug-susceptible TB, 73 no prior TB). We did not identify associations with bedaquiline-resistance, other than residence in Cape Town (OR 1.61, 1.19–2.20) and resistance to other drugs (fluoroquinolone resistance the strongest; OR 4.38, 3.20–5.96). In people initially bedaquiline-susceptible with a later isolate tested, 22% (45/201) gained resistance. Bedaquiline-resistance was most frequent in the Overberg region [14% (8, 23) of RR/MDR-TB]. 86% (2411/2799) of people had a successful linezolid pDST, with <1% (2/2411) resistant. All 128 people with repeat linezolid pDST remained susceptible.

**Conclusion:**

About one in ten people with RR/MDR-TB had bedaquiline-resistance, with half due to primary transmission. One in five people with RR/MDR-TB did not have bedaquiline DST done, highlighting care cascade gaps. Despite long treatment and sustained culture positivity, minimal linezolid resistance occurred.

## Introduction

Drug-resistant tuberculosis (DR-TB) is a global health threat. The World Health Organization (WHO) aims to reduce TB deaths and incidence by 95% and 90% respectively by 2035^1^. To achieve this, new and repurposed drugs like bedaquiline and linezolid are crucial to treat rifampicin-resistant (RR) and multidrug-resistant (MDR)-TB^2^.

South Africa was one of the first countries to make these drugs programmatically available, prioritising them for people with few treatment options before scale-up to all people with RR/MDR-TB^3,4^. The South African diagnostic cascade first involves the detection of TB and resistance to rifampicin and/or isoniazid using a nucleic acid amplification test. If RR/MDR-TB is identified, further drug susceptibility testing (DST) for fluoroquinolones (using Xpert MTB/XDR), bedaquiline and linezolid are done [using phenotypic (p)DST].

The global frequency of bedaquiline resistance amongst people with RR/MDR-TB is estimated at 6%, ranging from 2% in China to 19% in Moldova^5^. In South Africa, bedaquiline resistance has been documented at rates from 4% to, in people with sustained positivity during bedaquiline-based treatment, as high as 55%^5–8^. High linezolid resistance rates (33%) have been noted in people in whom linezolid-based treatment is failing^9^.

Resistance to bedaquiline and linezolid is influenced by overlapping risk factors, including previous RR/MDR-TB, adverse events, prior bedaquiline and clofazimine exposure, and a limited number of likely effective drugs, with bedaquiline resistance appearing more common in people with fluoroquinolone resistance^6,8,10^. Bedaquiline’s relatively long half-life may contribute to sub-therapeutic drug concentrations, promoting resistance acquisition^11^.

More data are needed on rates of resistance in programmatic contexts, and the extent of resistance gain among people initially susceptible. This would inform whom should be prioritised for DST to Group A drugs, which is phenotypic (slow), expensive, and technically demanding. Furthermore, the extent to which bedaquiline resistance is geographically heterogeneous in South Africa is unclear. Identifying hotspots could support targeted interventions, including improved surveillance, active case finding, and treatment support.

Using routine data from a centralised reference laboratory, we aimed to determine rates and correlates of bedaquiline-, linezolid- and fluoroquinolone-resistance in the Western Cape Province of South Africa. This was done to provide insights into resistance-emergence on a population-level; informing improved DST and treatment.

## Materials and Methods

### Setting

We retrospectively analysed people with at least RR/MDR-TB who submitted sputum 01/01/2023–31/12/2024 for diagnosis or DST at the National Health Laboratory Services (NHLS), Greenpoint, which conducts most government sector second-line pDST in the South African Western Cape Province.

### Data collection

We collected available demographic information (age, sex, HIV, submitting facility), routine results [Xpert MTB/RIF Ultra (Ultra; Cepheid, Sunnyvale, USA) for rifampicin susceptibility, Xpert MTB/XDR (Cepheid) for fluoroquinolone susceptibility], and bedaquiline and linezolid pDST results obtained using Mycobacterium Growth Indicator Tube 960 culture (MGIT960, Becton Dickinson, USA). We evaluated laboratory information system records for evidence of prior TB and drug susceptibility. In people with a fluoroquinolone, bedaquiline, and/or linezolid DST result, we collected results from the same DSTs from later specimens from the same episode. Repeat DST is not automatic and typically triggered by a clinical decision and/or sustained culture positivity.

### Analysis

We obtained sub-district (n = 36) and district (n = 6) RR/MDR-TB count data and analysed fluoroquinolone-, bedaquiline- and linezolid-resistance per 100 people with RR/MDR-TB with a resistant or susceptible result for the relevant second-line drug. We generated heatmaps using QGIS [v3.34.12-Prizren; https://qgis.org/] based on facility locations. For results from specialised centres (Brooklyn Chest Hospital, DP Marais), the referring facility location was used.

### Ethics

This study was approved by the Human Research Ethics Committee Division of Molecular and Human Genetics, Department of Biomedical Sciences at Stellenbosch University (S20/08/189, N09/11/296) and NHLS (PR2119347). Written informed consent was waived as anonymised data were collected retrospectively from routine care.

## Results

### Demographics and associates of initial bedaquiline resistance

The median (IQR) age was 36 years (28–45), with most people male (59%) and almost half (49%) of people living with HIV. People with RR/MDR-TB from the Cape Town metropolitan district were more likely to have bedaquiline-resistance [odds ratio (OR) 1.61 (95% confidence interval [CI], 1.19–2.20) vs. those from other districts], as were people with resistance to other second-line drugs, especially fluoroquinolones [35% (81/232) vs 11% (239/2191) in fluoroquinolone susceptible TB; OR 4.38 (3.20–5.96)] (Table 1).

### Initial drug susceptibility

#### Fluoroquinolones:

**Figure 1:**
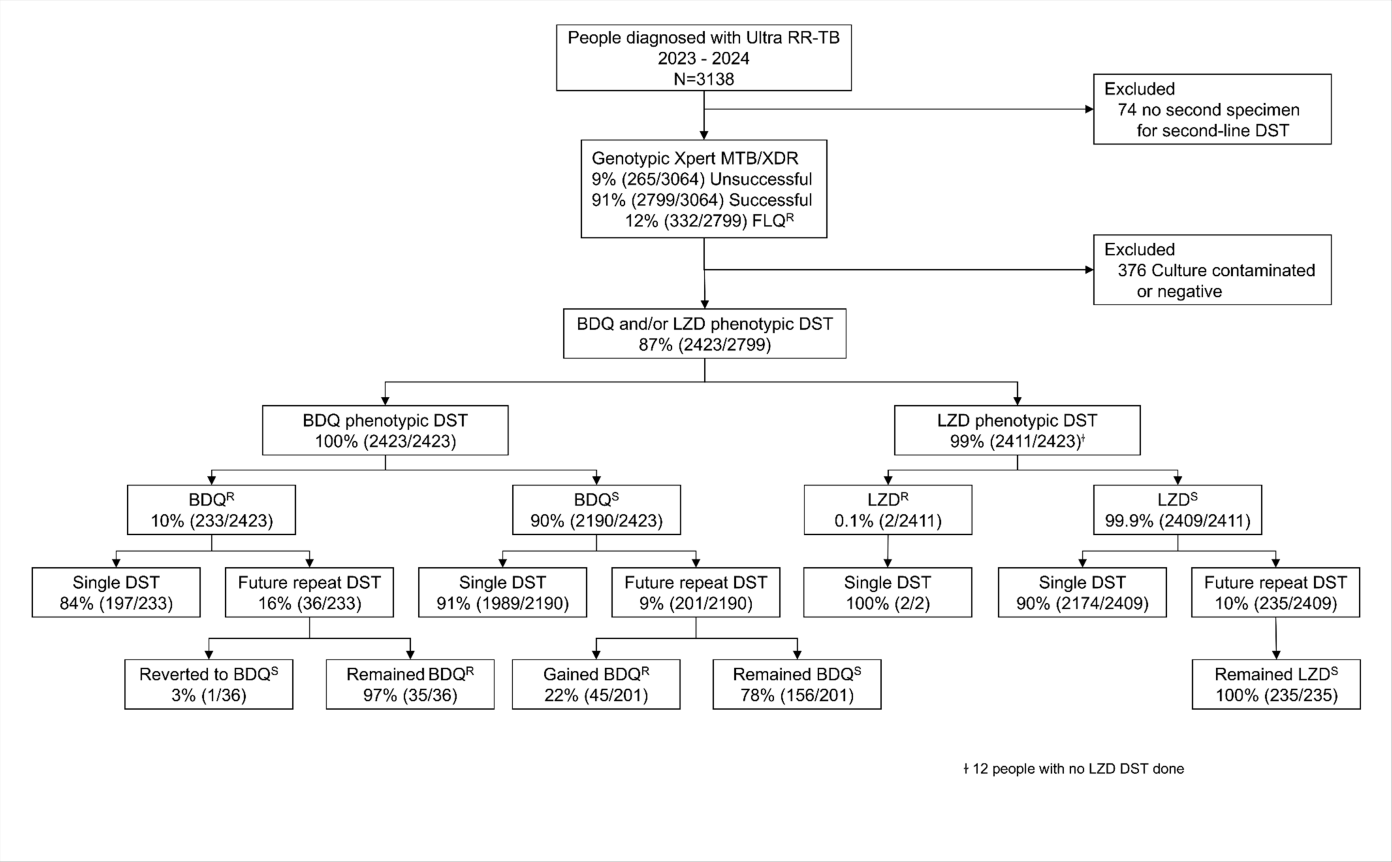
Study profile. We quantified the frequency of bedaquiline-resistant among people with RR/MDR-TB on respiratory specimens in a high-volume, programmatic laboratory over two years. We showed 12% of individuals were bedaquiline-resistant, with 22% people initially bedaquiline-susceptible and gaining resistance. Abbreviations: BDQ^R^, bedaquiline-resistant; BDQ^S^, bedaquiline-susceptible; BDQ^R^, bedaquiline-resistant; DST, drug susceptibility testing; FLQ^R^, fluoroquinolones-resistant; LZD^R^, linezolid-resistant; LZD^S^, linezolid-susceptible; RR-TB, rifampicin-resistant tuberculosis; Ultra, Xpert MTB/RIF Ultra.

Ninety-eight percent (3064/3138) of people with RR/MDR-TB had Xpert MTB/XDR attempted, 91% (2799/3064) had a susceptible or resistant result, and 13% (320/2799) were fluoroquinolone-resistant (Fig. 1).

#### Bedaquiline:

Overall, 77% (2423/3138) of people with RR/MDR-TB had results available, of which 12% (278/2423) were bedaquiline-resistant. 23% (715/3138) of people did not have susceptibility determined, with reasons including not culture-positive [90% (641/715); 543 culture-negative, 98 culture-contaminated] and no second specimen [10% (74/715)]. Eighty-three percent (232/278) of people with bedaquiline-resistance had their first isolate resistant, and of these, 51% (118/232) had no prior DR-TB, 19% (45/232) had prior drug-susceptible TB, and 32% (73/232) had never had TB before. Of people who were bedaquiline resistant and had a fluroquinolone DST result, 35% (81/232) were fluroquinolone-resistant and 65% (150/232) fluroquinolone-susceptible.

#### Linezolid:

Two of the 2411 people who successfully underwent pDST were linezolid-resistant (also fluoroquinolone- and bedaquiline-resistant).

### Susceptibility during treatment

#### Fluoroquinolones:

Three percent (12/401) of people susceptible at the start of their episode gained resistance with a median (IQR) time-to-resistance of seven months (6–12).

#### Bedaquiline:

**Figure 2:**
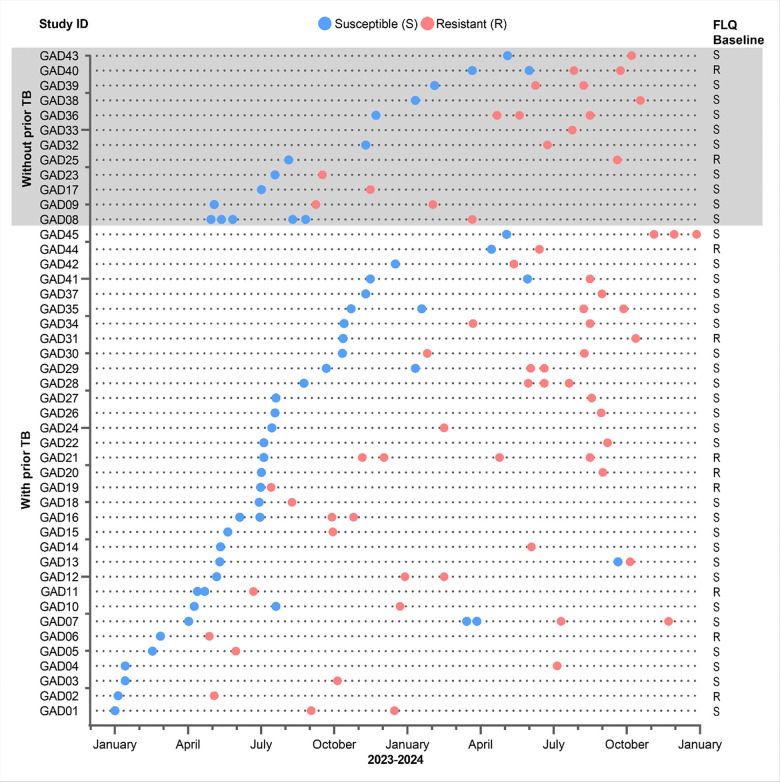
Timeline of bedaquiline resistance gain grouped by prior TB status: In people initially bedaquiline-susceptible in whom the programme repeated DST, 22% gained resistance, of which 30% of them had no evidence of a prior TB episode, and all were linezolid-susceptible. Blue circles represent susceptible, red circles represent resistant results, and the grey section colour represents people without prior TB. Abbreviations: DST, drug susceptibility testing; FLQ, fluoroquinolones; TB, tuberculosis.

Per-person data for bedaquiline resistance gain is in Fig. 2. Ten percent (237/2423) had a repeat bedaquiline pDST done [90% (2186/2423) no repeat DST], of which 15% (36/237) and 85% (201/237) were resistant and susceptible, respectively. Twenty-two percent (45/201) of people susceptible at the start of their episode gained resistance with a median (IQR) time-to-resistance of six months (4–10) and remained resistant after repeat DST. Seventy-three percent (33/45) had prior-TB, of which 30% (10/33) had baseline fluoroquinolone-resistance and 100% (33/33) had linezolid-susceptible.

#### Linezolid.

All 128 people susceptible repeat the start of their episode and with repeat DST remained susceptible over a median (IQR) of eight months (5–11).

### Resistance by distric

#### Resistance by sub-district is in Table 2.

##### Fluoroquinolone:

Per 100 RR/MDR-TB people with a fluoroquinolone-resistant or susceptible result resistance was most common in the West Coast at 15% (95% CI: 11, 20), followed by Cape Town Metropolitan at 13% (12, 15), Cape Winelands at 9% (6, 12), Overberg at 8% (4, 15), Central Karoo at 9% (3, 21), and Garden Route at 5% (3, 9).

##### Bedaquiline:

**Figure 3:**
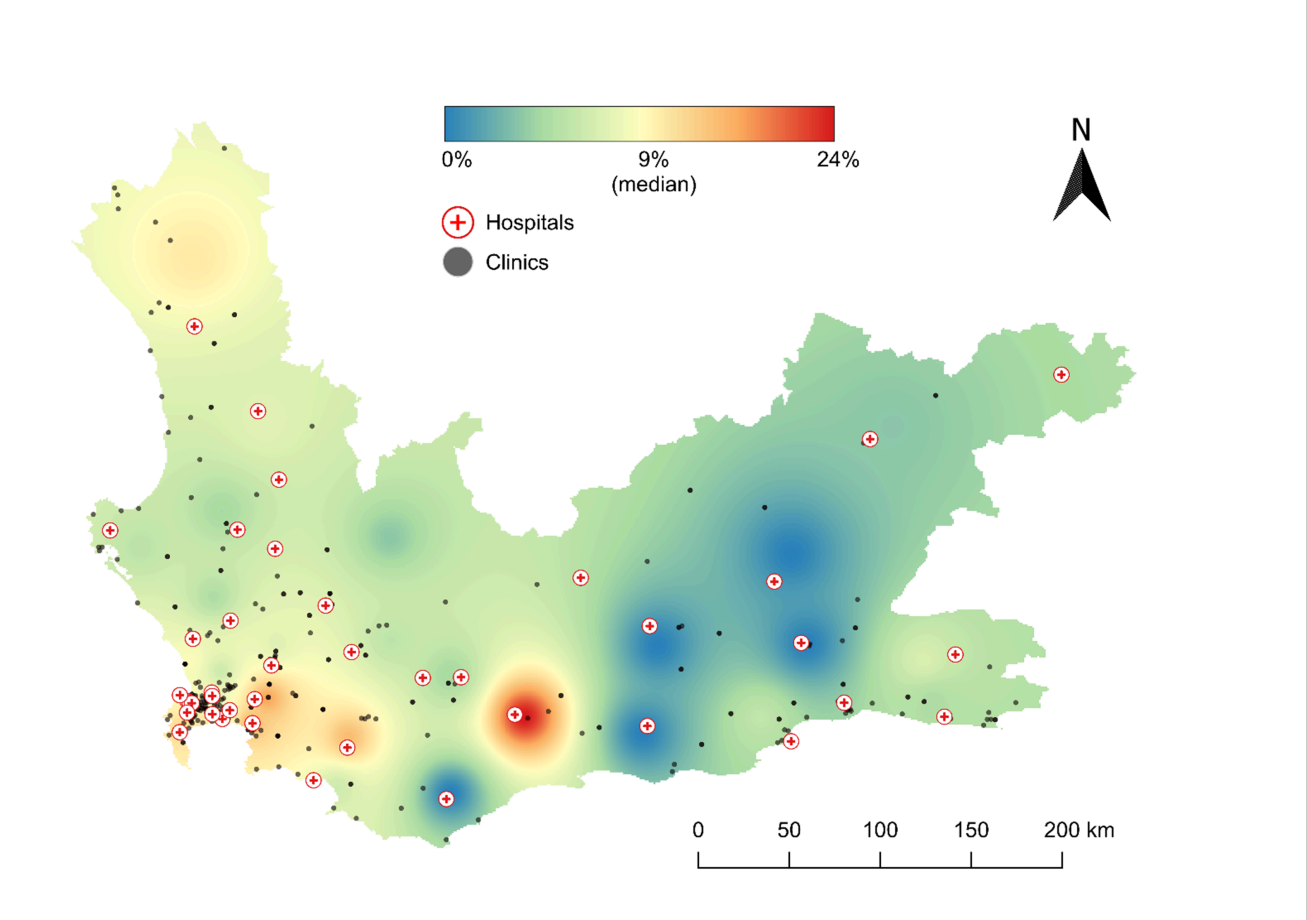
Spatial distribution of bedaquiline-resistance proportion per 100 RR/MDR-TB cases, aggregated by sub-district level of the Western Cape Province. Case counts concentrated in Overberg and Cape Town. Colours are broken by quantiles per 100 RR/MDR-TB cases. Black dots denote clinic locations, and red crosses within the red circle denote hospitals. The number of people with cases used to plot each drug-resistance estimate is in Table 2. Each colour and intensity represents the case count for each resistance to each drug.

Per 100 RR/MDR-TB people with a bedaquiline-resistant or susceptible result, resistance was most common in the Overberg at 14% (95%CI: 8, 23) (Fig. 3), followed by Cape Town Metropolitan at 13% (11, 15), Cape Winelands at 10% (7, 14), West Coast at 9% (6, 13), Garden Route at 7% (4, 11), and Central Karoo at 2% (0, 11).

## Discussion

Our key findings are, in people with RR/MDR-TB in the Western Cape Province: 1) 12% were bedaquiline-resistant, with rates highest in the Overberg district, and bedaquiline-resistance was associated fluoroquinolone resistance, 2) 51% of bedaquiline-resistance was in people without prior RR/MDR-TB who likely had no prior bedaquiline exposure, 3) in people with sustained culture positivity with programmatic follow-up DST, 22% gained bedaquiline-resistance, and 4) one in four people with RR/MDR-TB who should have a bedaquiline DST result did not have one. Lastly, 5) linezolid resistance was rare, even in people with long linezolid-based treatment and sustained culture-positivity. These findings demonstrate that routine diagnostic data can be used for surveillance to potentially inform targeted intervention strategies.

Rates of bedaquiline-resistance in this study exceeded earlier national estimates (4% from 2015–2019) and were comparable with a more recent report of 10%^7^. Reported prevalence across South Africa varies widely (4–55%), with the higher estimates largely reflecting data from difficult-to-treat populations with advanced drug-resistance ^5–7,12^. We detected most bedaquiline-resistance in Cape Town and the Overberg districts, which is comparable to previous studies in the Western Cape province that identified concentrated pockets of DR-TB, however, these did not include Group A drug resistance ^13,14^. Such hotspots should be targeted for intensified contact tracing, better upfront DST coverage in RR/MR-TB cases, and active surveillance to monitor resistance in real time. People with fluoroquinolone resistance were five times more likely to have bedaquiline-resistance, underscoring the importance of rapid fluoroquinolone DST to potentially prevent bedaquiline-resistance emergence.

Our findings suggest that one in two individuals with bedaquiline-resistant TB had no documented exposure to bedaquiline, and one in three had no evidence of prior TB, indicating primary transmission. Furthermore, 22% of initially susceptible individuals in whom the programme repeated pDST gained bedaquiline-resistance comparable to the 21% observed in individuals with prior bedaquiline exposure^7^. These findings highlight the vulnerability of current bedaquiline-based regimens to resistance and reinforce the need for rapid diagnostics and ongoing resistance monitoring.

Phenotypic DST was not done in 23% of people with RR/MDR-TB, because the second specimen was unavailable, culture-negative or -contaminated. This gap has significant programmatic implications, particularly in high-burden settings where empirical treatment (possibly a weaker background regimen) is often initiated without comprehensive DST. The clinical relevance of Ultra RR/MDR-TB, culture-negative results remains uncertain, potentially reflecting false-positives or early culture-negative TB^15,16^.

Linezolid-resistance was rare, even in people with sustained culture-positivity; similar to previously reported rates of 0.2–2% in South Africa^7^ <1% in Russia^17^. This finding reassures linezolid’s usage; however, challenges remain in programmatic use, with resistance detected in a third of high-risk cohorts, primarily in the Eastern Cape Province, South Africa^9^ and one in four patients for whom treatment failed in Moldova^18^. Surveillance is crucial to identify and monitor the emergence of linezolid resistance using available rapid DST (such as the LiquidArray MTB-XDR^19^).

Our study has strengths and limitations. Phenotypic DST data were available for bedaquiline and linezolid, but genomic data were not. Despite these, our findings reveal a sizeable proportion and spatial variation of drug resistance. We used the most recent bedaquiline-susceptible RR/MDR-TB isolate to assess resistance gain; however, earlier susceptible isolates may exist, which could affect the time-to-resistance-gain estimate. Spatial analysis was based on facility locations rather than households, assuming people seek care near their homes. Our data excluded the private sector, potentially underestimating the case.

In summary, about one in ten people with RR/MDR-TB had bedaquiline resistance, with about half due to primary transmission. Over a quarter of people did not have bedaquiline susceptibility determined, highlighting a care cascade gap. We identified minimal linezolid-resistance even after long-term treatment with sustained culture-positivity, while bedaquiline-resistance hotspots and significant bedaquiline-resistance gain reinforce the need for surveillance, targeted interventions, and rapid molecular DST.

## Figures and Tables

**Table 1:** Demographic and clinical characteristics of people with RR/MDR-TB, stratified by initial bedaquiline susceptibility. People with RR/MDR-TB and initial bedaquiline resistance were more likely to live in Cape Town and have co-resistance to fluoroquinolones, isoniazid, ethionamide, and amikacin. Data are n/N (%) or median (IQR) unless otherwise indicated.

Variables	Overall RR/MDR-TB (n = 3138)	Initial bedaquiline-susceptibility result^#^		OR for baseline bedaquiline resistance (95% CI), p-value

		Resistant (n = 232)	Susceptible (n = 2191)	
Age, years	38 (29–47)	34 (28–41)	36 (28–46)	1.01 (1.01, 1.02), p = 0.060

Male	1847/3138 (59)	137/231 (59)	1297/2173 (60)	0.98 (0.74, 1.31), p = 0.911

HIV Positive	429/886 (48)	63/138 (46)	608/1232 (49)	0.86 (0.60, 1.25), p = 0.410

Live in Cape Town metropole	1923/3138 (61)	166/232 (72)	1336/2191 (61)	1.61 (1.19, 2.20), **p < 0.0016**

Ultra information				
TB-specific C_T_ values	16.3 (16.2–16.8)	16.2 (16.2–16.6)	16.2 (16.2–16.9)	0.99 (0.90, 1.10), p = 0.867
Semi-quantitative load	88/1516 (6)	10/144 (7)	79/1373 (6)	1.22 (0.55, 2.44), p = 0.563
Very low	278/1516 (18)	20/144 (14)	258/1373 (19)	0.70 (0.40, 1.15), p = 0.148
Low	342/1516 (23)	28/144 (19)	314/1373 (23)	0.81 (0.51, 1.26), p = 0.394
Medium High	808/1516 (53)	85/144 (59)(53)	723/1373	1.30 (0.90, 1.87), p = 0.145

Resistance				
Fluoroquinolone	332/2799 (12)	81/232 (35)	239/2191 (11)	4.38 (3.20, 5.96), **p < 0.0001**
Isoniazid	1738/2799 (62)	199/232 (86)	1540/2191 (70)	2.55 (1.73, 3.85), **p < 0.0001**
Ethionamide	1120/2799 (40)	132/218 (61)	887/2097 (42)	2.09 (1.56, 2.82), **p < 0.0001**
Amikacin	110/2799 (4)	25/230 (11)	80/2191 (4)	3.21 (1.92, 5.23), **p < 0.0001**

Abbreviations: CI, confidence interval; IQR, interquartile range; OR, odds ratio; RR-TB, rifampicin-resistant tuberculosis; Ultra, Xpert MTB/RIF Ultra.

# Missing data: bedaquiline (n = 1), age (n = 16); sex (n = 19); HIV (n = 1053), only two phenotypic linezolid-resistant cases, C_T_ values (n = 1837)

**Table 2:** Drug resistance per 100 RR/MDR-TB cases by drug and sub-districts. Swellendam sub-district within the Overberg had the highest bedaquiline-resistant rate, while Prince Albert within the Central Karoo had the highest fluoroquinolone-resistant rate per 100 RR/MDR-TB people. Data are n/N (relative frequency) unless otherwise indicated.

District	Sub-district^#^	Fluoroquinolones-DST		Bedaquiline-DST	
		Number of people with resistant results among people tested	Resistance rate per 100 RR/MDR-TB (95% CI)	Number of people with resistant results among people tested	Resistance rate per 100 RR/MDR-TB (95% CI)
Central Karoo	Beaufort West	1/28 (0.04)	4 (0, 18)	1/22 (0.05)	5 (0, 23)
	Langeberg	0/25 (0.00)	0 (0, 14)	1/17 (0.06)	6 (0, 29)
	Prince Albert	4/12 (0.33)	33 (10, 65)	0/9 (0.00)	0 (0, 34)
Cape Town Metropolitan	Eastern	40/276 (0.14)	14 (11, 19)	38/209 (0.18)	18 (13, 24)
	Khayelitsha	28/220 (0.13)	13 (9, 18)	19/177 (0.11)	11 (7, 16)
	Klipfontein	32/207 (0.15)	15 (11, 21)	28/192 (0.15)	15 (10, 20)
	Mitchells Plain	25/217 (0.12)	12 (8, 17)	31/207 (0.15)	15 (10, 21)
	Northern	7/91 (0.08)	8 (3, 15)	6/70 (0.09)	9 (3, 18)
	Southern	40/152 (0.26)	26 (20, 34)	26/174 (0.15)	15 (10, 21)
	Tygerberg	31/316 (0.10)	10 (7, 14)	19/248 (0.08)	8 (5, 12)
	Western	25/230 (0.11)	11 (7, 16)	27/230 (0.12)	12 (8, 17)
Cape Winelands	Breede Valley	4/105 (0.04)	4 (1, 9)	6/80 (0.08)	8 (3, 16)
	Drakenstein	20/169 (0.12)	12 (7, 18)	14/141 (0.1)	10 (6, 16)
	Stellenbosch	6/53 (0.11)	11 (4, 23)	8/44 (0.18)	18 (8, 33)
	Witzenberg	3/51 (0.06)	6 (1, 16)	2/43 (0.05)	5 (1, 16)
Garden Route	George	6/123 (0.05)	5 (2, 10)	10/106 (0.09)	9 (5, 17)
	Hessequa	1/5 (0.20)	20 (1, 72)	0/4 (0.00)	0 (0, 60)
	Kannaland	0/12 (0.00)	0 (0, 26)	0/9 (0.00)	0 (0, 34)
	Knysna	0/29 (0.00)	0 (0, 12)	2/31 (0.06)	6 (1, 21)
	Mossel Bay	1/19 (0.05)	5 (0, 26)	1/13 (0.08)	8 (0, 36)
	Oudtshoorn	2/33 (0.06)	6 (1, 20)	0/30 (0.00)	0 (0, 12)
Overberg	Cape Agulhas	1/14 (0.07)	7 (0, 34)	0/12 (0.00)	0 (0, 26)
	Overstrand	2/25 (0.08)	8 (1, 26)	2/23 (0.09)	9 (1, 28)
	Swellendam	4/21 (0.19)	19 (5, 42)	5/21 (0.24)	24 (8, 47)
	Theewaterskloof	3/60 (0.05)	5 (1, 14)	7/42 (0.17)	17 (7, 31)
West Coast	Bergrivier	1/20 (0.05)	5 (0, 25)	1/17 (0.06)	6 (0, 29)
	Cederberg	9/41 (0.22)	22 (11, 38)	4/40 (0.10)	10 (3, 24)
	Matzikama	18/94 (0.19)	19 (12, 29)	11/82 (0.13)	13 (7, 23)
	Saldanha	9/77 (0.12)	12 (5, 21)	5/68 (0.07)	7 (2, 16)
	Swartland	9/74 (0.12)	12 (6, 22)	4/62 (0.06)	6 (2, 16)

Abbreviations: CI, confidence interval; RR, rifampicin-resistant; TB, tuberculosis.

# Missing data: sub-district (n = 2, Bitou, Langeberg)
